# Ares-GT: Design of guide RNAs targeting multiple genes for CRISPR-Cas experiments

**DOI:** 10.1371/journal.pone.0241001

**Published:** 2020-10-21

**Authors:** Eugenio Gómez Minguet

**Affiliations:** Independent Researcher, Manises (Valencia), Spain; University of Leeds, UNITED KINGDOM

## Abstract

Guide RNA design for CRISPR genome editing of gene families is a challenging task as usually good candidate sgRNAs are tagged with low scores precisely because they match several locations in the genome, thus time-consuming manual evaluation of targets is required. To address this issues, I have developed ARES-GT, a Python local command line tool compatible with any operative system. ARES-GT allows the selection of candidate sgRNAs that match multiple input query sequences, in addition of candidate sgRNAs that specifically match each query sequence. It also contemplates the use of unmapped contigs apart from complete genomes thus allowing the use of any genome provided by user and being able to handle intraspecies allelic variability and individual polymorphisms. ARES-GT is available at GitHub (https://github.com/eugomin/ARES-GT.git).

## Introduction

The design of optimal single guide RNAs (sgRNAs) is a critical step in CRISPR/Cas genome editing, and it must ensure specificity and minimize the possibility of offtarget mutations. Although good online tools are available for identification of CRISPR DNA targets, which have popularized genome editing, their use is limited to a restricted list of genomes [1–6], sometimes corresponding to less than ten species [7, 8]. Even Breaking-Cas [9], a free online tool which currently offers more than 1600 genomes, lacks the flexibility to easily incorporate unpublished genomes or contemplate genomes of populations with allelic variants -an issue partially addressed by AlleleAnalyzer for the human genome [10]. Several command-line tools present more flexibility incorporating any genome provided by users, like sgRNA-cas9 [11] or CRISPRseek [12]. However, an additional problem posed by the design of sgRNAs targeting gene families is that good candidate sgRNAs can be tagged with low scores precisely because they match several locations in the genome, thus time-consuming manual evaluation of targets is required. To address this issue, I have developed ARES-GT, a local command line tool in Python programming language.

## Methods

### ARES-GT

ARES-GT is written in python programming language (https://www.python.org/) so it can be runned in any operative system. The software is available at GitHub (https://github.com/eugomin/ARES-GT.git): version 2.0 is a python2.7 version while version 2.0.1 is updated to python3.8. In addition of sys and re modules, ARES-GT also requires the third-party regex module (https://pypi.org/project/regex/).

Complete analysis presented in this work were performed in minimum 3 hours and maximum 12 hours, depending of the analysis, in a Linux server running Ubuntu 18.04 LTS with Intel Xeon 2.0 GHz processor and 32 GB RAM. When option “OR” is selected (so only analysis of candidates matching several query sequences), the same analysis were performed in 15 min or less. Running time directly depends on the number of query sequences, genome size and selected parameters.

### Genome sequences

Arabidopsis reference genome (Col-0) were obtained from TAIR (www.arabidopsis.org). Good quality genome assemblies of seven *A*. *thaliana* accessions (*An-1*, *C24*, *Cvi*, *Eri*, *Kyo*, *Ler* and *Sha*) [13] were downloaded from Arabidopsis 1001 genomes project (https://1001genomes.org/), and *Cardamine hirsuta* genome from its genetic and genomic resource (http://chi.mpipz.mpg.de/index.html). All sequences of CBF genes are available in S1 File.

### CBF genes

Genomic sequences of *Arabidopsis thaliana* CBF genes (AtCBFs) were obtained from TAIR (https://www.arabidopsis.org/), corresponding to Col-0 TAIR v10. Genomic sequences of AtCBFs homologs in *C*. *hirsuta* were identified by BLAST in the *C*. *hirsuta* genetic and genomic resource (http://chi.mpipz.mpg.de/index.html) using the AtCBFs protein sequences and supported by alignment with ClustalX2 [14]. Ecotype specific genomic sequence of each CBF gene were retrived using the genomic coordinates from ARES-GT results using AtCBFs (Col-0).

## Results

### Identification of CRISPR targets candidates

The high sequence similarity shared in gene families increase the possibility of also sharing CRISPR targets, both with perfect match or with few mismatches. While this is especially interesting for targeting multiple members of the same family, they are usually discarded or evaluated with low scores. Similarly to other available software, ARES-GT starts with the identification of all candidate guide RNAs in query sequences and then the reference genome is used to find possible offtargets, but an additional step is added to evaluate which guide sequences match several query sequences.

Offtargets evaluation is based in a mismatch criteria. It has been reported that the specificity of both Cas9 and Cas12a is particularly sensitive to mismatches in the PAM proximal sequence (on an 11- and 8-nucleotide stretch for Cas9 and Cas12a, respectively), named “seed” [15–18]. Mismatches in the seed sequence has a critical impact into cleavage efficiency on DNA target, and it is unlikely that seed sequences with 2 or more mismatches cause real offtargets *in vivo*. *S*equence composition and the number and distribution of mismatches also affects cleavage efficiency [15]. Therefore the ARES-GT algorithm discards possible offtargets using as criterium the presence of 2 or more mismatches in the seed sequence, while the user defines the threshold criterium out of seed sequence. In addition, the user must also indicate whether a “NAG” PAM, which Cas9 can recognise though with lower efficiency [15], must be taken into account when evaluating possible Cas9 offtargets.

ARES-GT can identify targets of the two most widely used CRISPR enzymes (Cas9 and Cas12a/Cpf1) and evaluates possible offtargets in a user-provided reference genome, including non assembled contigs and unpublished genomes from any species. A list is generated with the best candidates (those with no offtargets based on parameters selected by user) and, if multiple query genes from the same family are targeted, the list includes sgRNAs that match more than one of them. Detailed information for each possible target is also provided, including an alignment with the possible offtargets. ARES-GT have been already used successfully in *Arabidopsis*, tomato and rice while under development [19, 20].

### Design of guide RNA matching multiple CBF genes

**Fig 1:**
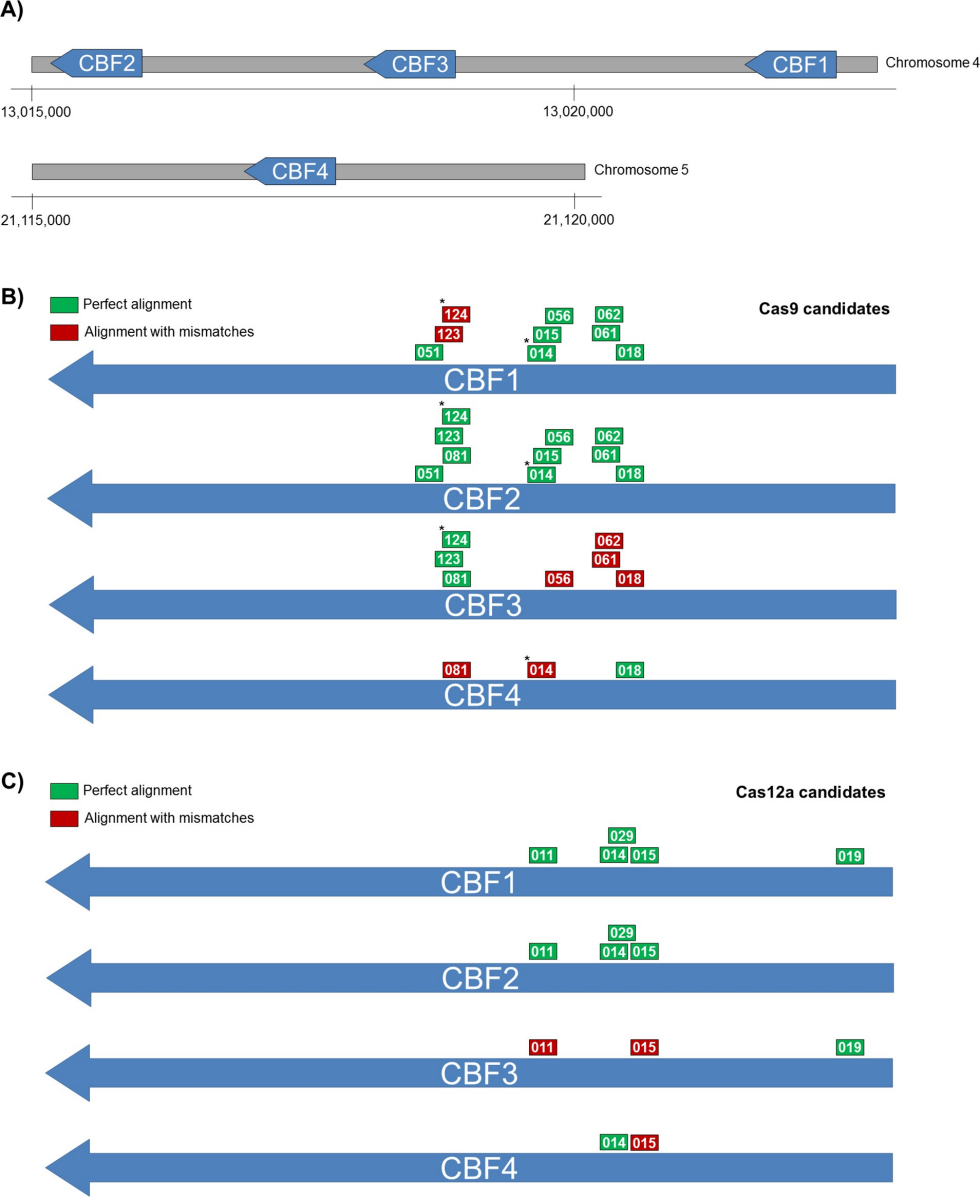
sgRNS targets in CBF genes. A) Genomic distribution of CBF genes in Arabiopsis thaliana chromosomes 4 and 5. Location of Cas9 (B) and Cas12a (C) candidates with multiple CBF gene targets. (*) Asterisk marks candidates corresponding with previously reported sgRNAs (Cho et al., 2017).

As a proof of concept, I have choosen the C-repeat/DRE-Binding Factor (CBF) gene family of plant transcription factors to test the various novelties implemented in ARES-GT. Among the four members identified in *Arabidopsis thaliana*, three of them–*AtCBF1*, *AtCBF2* and *AtCBF3–*, have been implicated in the response to cold temperatures, while *AtCBF4* has been implicated in the response to drought [21, 22]. The first three members of this family are closely located in less than 8 Kb in chromosome 4 (Fig 1A), making extremely difficult to obtain a triple mutant by classical crossing strategy. This has been recently achieved by CRISPR/Cas9-induced mutagenesis [23] using two sgRNAs that the authors selected by manual evaluation of sequence alignments, manual selection of candidates, and specificity verification with CRISPR-P [1]. I used the *A*. *thaliana* genomic coding sequences (TAIR v10) of the four *CBF* genes as a multiple query in ARES-GT, to search for candidate sgRNAs using both Cas9 and Cas12a. A total of 96 and 34 unique specific targets matching only one location in the genome and with no predicted offtargets were found for each the four genes, using Cas9 and Cas12a, respectively. More interestingly, the program also listed 13 candidates for Cas9 and 10 candidates for Cas12a that match multiple *CBF* genes (Tables 1 and 2). In total, 10 Cas9 and 5 Cas12a candidates were identified that match more than one *CBF* gene and did not present any offtarget outside *CBF* genes (Fig 1B and 1C). Among them were included the two sequences previously reported [23], corresponding to Cas9CBF1_015 and Cas9CBF2_124 in this work.

**Table 1:** Multiple targets Cas9 candidates for *AtCBF* genes. All possible genome targets and offtargets (with ARES-GT thresholds: L0 = 4 and L1 = 3) of each candidate are listed with indication of genome coordinates (TAIR v10) and whether it corresponds to a *CBF* gene. In alignments, black boxes mark mismatches and a space separates PAM (NGG or NAG) from sequence. Differences in the “N” position in the PAM are not marked.

Candidate ID	Targets + Offtargets (L0 = 4, L1 = 3)					
*A*. *thaliana*	*Gene*	*chrom*	*start*	*end*	*sense*	*sequence*
Cas9AtCBF1_014	*AtCBF2*	4	13015820	13015842	+	AGCACGAGCTGCCATCTCAG CGG
	*AtCBF1*	4	13022305	13022327	+	AGCACGAGCTGCCATCTCAG CGG
	*AtCBF3*	4	13018737	13018759	+	AGCTCGAGCTGCCATCTCAG CGG
Cas9AtCBF1_015	*AtCBF2*	4	13015825	13015847	+	GAGCTGCCATCTCAGCGGTT TGG
	*AtCBF1*	4	13022310	13022332	+	GAGCTGCCATCTCAGCGGTT TGG
Cas9AtCBF1_018	*AtCBF2*	4	13015920	13015942	+	TGACGAACTCCTCTGTAAAT TGG
	*AtCBF1*	4	13022405	13022427	+	TGACGAACTCCTCTGTAAAT TGG
	*AtCBF4*	5	21117612	21117634	+	TGACGAACTCCTCTGTAAAT CGG
	*AtCBF3*	4	13018837	13018859	+	CGACGAACTCCTCTGTATAT TGG
Cas9AtCBF1_019	*AtCBF2*	4	13015921	13015943	+	GACGAACTCCTCTGTAAATT GGG
	*AtCBF1*	4	13022406	13022428	+	GACGAACTCCTCTGTAAATT GGG
	----	1	1597274	1597296	+	CACAATCTCCTCTGTAAATT CAG
	*AtCBF3*	4	13018838	13018860	+	GACGAACTCCTCTGTATATT GGG
Cas9AtCBF1_051	*AtCBF2*	4	13015738	13015760	-	CCG GGATTCGTAGCCGCCAAGCC
	*AtCBF1*	4	13022223	13022245	-	CCG GGATTCGTAGCCGCCAAGCC
Cas9AtCBF1_056	*AtCBF2*	4	13015831	13015853	-	CCA TCTCAGCGGTTTGGAAAGTC
	*AtCBF1*	4	13022316	13022338	-	CCA TCTCAGCGGTTTGGAAAGTC
	*AtCBF3*	4	13018748	13018770	-	CCA TCTCAGCGGTTTGAAATGTT
Cas9AtCBF1_061	*AtCBF2*	4	13015900	13015922	-	CCC ACTTACCGGAGTTTCTTTGA
	*AtCBF1*	4	13022385	13022407	-	CCC ACTTACCGGAGTTTCTTTGA
	*AtCBF3*	4	13018817	13018839	-	CCC ACTTACCGGAGTTTCTCCGA
Cas9AtCBF1_062	*AtCBF2*	4	13015901	13015923	-	CCA CTTACCGGAGTTTCTTTGAC
	*AtCBF1*	4	13022386	13022408	-	CCA CTTACCGGAGTTTCTTTGAC
	*AtCBF3*	4	13018818	13018840	-	CCA CTTACCGGAGTTTCTCCGAC
Cas9AtCBF1_063	*AtCBF2*	4	13015908	13015930	-	CCG GAGTTTCTTTGACGAACTCC
	*AtCBF1*	4	13022393	13022415	-	CCG GAGTTTCTTTGACGAACTCC
	----	2	6123419	6123441	-	CCC GACTTTCTTTGAAGAACTCC
Cas9AtCBF1_064	*AtCBF2*	4	13015929	13015951	-	CCT CTGTAAATTGGGTGACGAGT
	*AtCBF1*	4	13022414	13022436	-	CCT CTGTAAATTGGGTGACGAGT
	*AtCBF3*	4	13018846	13018868	-	CCT CTGTATATTGGGTGACGAGT
	----	1	4290740	4290762	-	CCT CTGTAAACTGGGTGACGTGT
	----	1	23368054	23368076	-	CCT CTGTAGATTGGGTGACGTGT
	*AtCBF4*	5	21117621	21117643	-	CCT CTGTAAATCGGATGACGTGT
Cas9AtCBF2_081	*AtCBF2*	4	13015760	13015782	+	CGAGTCAGCGAAATTGAGAC AGG
	*AtCBF3*	4	13018677	13018699	+	CGAGTCAGCGAAATTGAGAC AGG
	*AtCBF4*	5	21117452	21117474	+	AGAATCAGCGAAATTGAGAC AAG
Cas9AtCBF2_123	*AtCBF2*	4	13015754	13015776	-	CCA AGCCGAGTCAGCGAAATTGA
	*AtCBF3*	4	13018671	13018693	-	CCA AGCCGAGTCAGCGAAATTGA
	*AtCBF1*	4	13022239	13022261	-	CCA AGCCGAGTCAGCGAAGTTGA
Cas9AtCBF2_124	*AtCBF2*	4	13015759	13015781	-	CCG AGTCAGCGAAATTGAGACAG
	*AtCBF3*	4	13018676	13018698	-	CCG AGTCAGCGAAATTGAGACAG
	*AtCBF1*	4	13022244	13022266	-	CCG AGTCAGCGAAGTTGAGACAT

**Table 2:** Multiple targets Cas12a candidates for *AtCBF* genes. All possible genome targets and offtargets (with ARES-GT thresholds: L0 = 4 and L1 = 3) of each candidate are listed with indication of genome coordinates (TAIR v10) and whether it corresponds to a *CBF* gene. In alignments, black boxes mark mismatches and a space separates PAM (TTTN) from sequence. Differences in the “N” position in the PAM are not marked.

Candidate ID	Targets + Offtargets (L0 = 4, L1 = 3)					
*A*. *thaliana*	*Gene*	*chrom*	*start*	*end*	*sense*	*sequence*
Cas12aAtCBF1_011	*AtCBF2*	4	13015814	13015837	-	GCTGCCATCTCAGCGGTTTG GAAA
	*AtCBF1*	4	13022299	13022322	-	GCTGCCATCTCAGCGGTTTG GAAA
Cas12aAtCBF1_012	*AtCBF2*	4	13015827	13015850	-	CGGTTTGGAAAGTCCCGAGC CAAA
	*AtCBF1*	4	13022312	13022335	-	CGGTTTGGAAAGTCCCGAGC CAAA
	----	1	27242286	27242310	+	TTTG GCTCGGGACTTTCAACACAG
	----	3	8296023	8296047	+	TTTG GCTCGGGACGTTCGAAAGCG
	----	5	17806910	17806934	+	TTTG GCTCGGGACATTCGACACGG
	----	5	21618544	21618567	-	CCGTCTCAAAAGTCCCGAGC CAAA
	----	4	7932903	7932927	+	TTTG GCTCGGCACTTTTGAAACCG
	----	4	10190722	10190745	-	CAGTTTGGAACGTTCCGAGC CAAA
	*AtCBF3*	4	13018744	13018767	-	CGGTTTGAAATGTTCCGAGC CAAA
Cas12aAtCBF1_014	*AtCBF2*	4	13015902	13015925	-	TTCTTTGACGAACTCCTCTG TAAA
	*AtCBF1*	4	13022387	13022410	-	TTCTTTGACGAACTCCTCTG TAAA
	*AtCBF4*	5	21117594	21117617	-	TCCTCTGACGAACTCCTCTG TAAA
Cas12aAtCBF1_015	*AtCBF2*	4	13015924	13015947	-	AATTGGGTGACGAGTCTCAC GAAA
	*AtCBF1*	4	13022409	13022432	-	AATTGGGTGACGAGTCTCAC GAAA
	*AtCBF3*	4	13018841	13018864	-	TATTGGGTGACGAGTCTCAC GAAA
	*AtCBF4*	5	21117616	21117639	-	AATCGGATGACGTGTCTCAC GAAA
Cas12aAtCBF1_017	*AtCBF2*	4	13016031	13016054	-	AATCGGAGCCAAACATTTCA GAAA
	*AtCBF3*	4	13018948	13018971	-	AATCGGAGCCAAACATTTCA GAAA
	*AtCBF1*	4	13022507	13022530	-	AATCGGAGCCAAACATTTCA GAAA
	----	1	8279033	8279056	-	AATCAGAGCCTAACACTTCA AAAA
	----	3	9399469	9399493	+	TTTA TGAAGTGTTTGGTTCCTATT
Cas12aAtCBF1_018	*AtCBF2*	4	13016032	13016055	-	ATCGGAGCCAAACATTTCAG AAAA
	*AtCBF3*	4	13018949	13018972	-	ATCGGAGCCAAACATTTCAG AAAA
	*AtCBF1*	4	13022508	13022531	-	ATCGGAGCCAAACATTTCAG AAAA
	----	1	9505057	9505081	+	TTTG CTGAAATGGTTGCCTCTAAT
Cas12aAtCBF1_019	*AtCBF3*	4	13018950	13018973	-	TCGGAGCCAAACATTTCAGA AAAA
	*AtCBF1*	4	13022509	13022532	-	TCGGAGCCAAACATTTCAGA AAAA
Cas12aAtCBF1_024	*AtCBF2*	4	13015842	13015865	+	TTTG GAAAGTCCCGAGCCAAATCC
	*AtCBF1*	4	13022327	13022350	+	TTTG GAAAGTCCCGAGCCAAATCC
	----	3	8296020	8296043	-	GGGTTTGGCTCGGGACGTTC GAAA
Cas12aAtCBF1_028	*AtCBF2*	4	13015913	13015936	+	TTTC TTTGACGAACTCCTCTGTAA
	*AtCBF1*	4	13022398	13022421	+	TTTC TTTGACGAACTCCTCTGTAA
	----	5	16311156	16311179	+	TTTT TTTGACGAATTTCTCTGTGG
Cas12aAtCBF1_029	*AtCBF2*	4	13015917	13015940	+	TTTG ACGAACTCCTCTGTAAATTG
	*AtCBF1*	4	13022402	13022425	+	TTTG ACGAACTCCTCTGTAAATTG

To test that AREST-GT can work with any user-provided genome, including unmapped contigs, I selected the first version of the genome of *Cardamine hirsuta* [24]. The available genome sequence spans over its 8 chromosomes, but also contains 622 unmapped contigs in addition to chloroplast and mithocondria genomes. The sequence information was downloaded and used locally with ARES-GT for searching CRISPR targets in the four *C*. *hirsuta CBF* homologous genes. In addition to unique specific targets (86 for Cas9 and 28 for Cas12a), 10 candidate sgRNAs for Cas9 and 3 for Cas12a were identified that perfectly match *ChCBF1* and *ChCBF2* (Table 3). Taking into account possible offtargets, only 5 and 3 sequences for Cas9 and Cas12a, respectively, are relyable candidate sgRNAs targeting only *ChCBF* family genes. For instance, Cas9ChCBF1_044 perfectly matches *ChCBF1* and *ChCBF2*, and it also matches *ChCBF3* with one mismatch.

**Table 3:** Multiple targets Cas9 and Cas12a candidates for *ChCBF* genes. All possible genome targets and offtargets (with ARES-GT thresholds: L0 = 4 and L1 = 3) of each candidate are listed with indication of genome coordinates (*Cardamine hirsuta* v1.0) and whether it corresponds to a *CBF* gene. In alignments, black boxes mark mismatches and a space separates PAM (NGG/NAG or TTTN) from sequence. Differences in the “N” position in the PAM are not marked.

Candidate ID	Targets + Offtargets (L0 = 4, L1 =3)					
*C*. *hirsuta*	*Gene*	*chrom*	*start*	*end*	*sense*	*sequence*
Cas9ChCBF1_004	ChCBF2	4	6514798	6514820	+	AGCTGTCCCAAGAAACCAGC TGG
	ChCBF1	7	17908883	17908905	-	CCG GCTGGTTTCTTGGGACAGCT
Cas9ChCBF1_010	ChCBF2	4	6514878	6514900	+	CTCCGGTAAGTGGGTGTGTG AGG
	ChCBF1	7	17908803	17908825	-	CCT CACACACCCACTTACCGGAGE
Cas9ChCBF1_018	ChCBF2	4	6514910	6514932	+	CAAACAAGAAATCTAGGATT TGG
	ChCBF1	7	17908771	17908793	-	CCA AATCCTAGATTTCTTGTTTG
	ChCBF3	8	13812274	13812296	-	CCA AATCCTCGATTTCTTGTTAG
	----	5	18638271	18638293	-	CTT AATCCTACATTTGTAGTTTG
	----	5	21152837	21152859	-	CTT AATCCTACATTTCTGGTTTT
Cas9ChCBF1_013	ChCBF2	4	6514915	6514937	+	AAGAAATCTAGGATTTGGCT TGG
	ChCBF1	7	17908766	17908788	-	CCG AGCCAAATCCTAGATTTCTT
	----	8	18333140	18333162	-	CCA AGCCAAATCCTAGAACCCTT
	----	1	5556241	5556263	+	AGGAAACGGAGGATTTGGCT TGG
	----	1	370416	370438	+	AAAAAATCTCGGATTTGGCT CGG
	ChCBF3	8	13812269	13812291	-	CCT AACCAAATCCTCGATTTCTT
Cas9ChCBF1_033	ChCBF2	4	6515264	6515286	+	TGCCGCCTCCGTCCGTACAA TGG
	ChCBF1	7	17908390	17908412	-	CCA TTGTACGGACGGAGGCGGCA
	NSCAFA.	444	2316	2338	+	CGCCGCCACCGTCCGTACAC CGG
Cas9ChCBF1_036	ChCBF2	4	6514793	6514815	-	CCG TGAGCTGTCCCAAGAAACCA
	ChCBF1	7	17908888	17908910	+	TGGTTTCTTGGGACAGCTCA CGG
Cas9ChCBF1_043	ChCBF2	4	6514880	6514902	-	CCG GTAAGTGGGTGTGTGAGGTA
	ChCBF1	7	17908801	17908823	+	TACCTCACACACCCACTTAC CGG
Cas9ChCBF1_044	ChCBF2	4	6514909	6514931	-	CCA AACAAGAAATCTAGGATTTG
	ChCBF1	7	17908772	17908794	+	CAAATCCTAGATTTCTTGTT TGG
	ChCBF3	8	13812275	13812297	+	CAAATCCTCGATTTCTTGTT AGG
Cas9ChCBF1_056	ChCBF2	4	6515266	6515288	-	CCG CCTCCGTCCGTACAATGGAA
	ChCBF1	7	17908388	17908410	+	TTCCATTGTACGGACGGAGG CGG
	----	2	8347578	8347600	+	GGCCAGAGTACGGACGGAGG AGG
Cas9ChCBF1_057	ChCBF2	4	6515269	6515291	-	CCT CCGTCCGTACAATGGAATCA
	ChCBF1	7	17908385	17908407	+	TGATTCCATTGTACGGACGG AGG
	----	1	17089187	17089209	+	TGGTCCGGTTGTACGGACGG CGG
	----	5	5225681	5225703	-	CCA CCGTCCGTACACTGGATTAT
Cas21aChCBF1_018	ChCBF2	4	6514830	6514853	+	TTTC GTGAGACTCGTCACCCAATT
	ChCBF1	7	17908848	17908871	-	AATTGGGTGACGAGTCTCAC GAAA
	ChCBF3	8	13812351	13812374	-	AATCGGATGACGTGTCTCAC GAAA
Cas21aChCBF1_029	ChCBF2	4	6515260	6515283	+	TTTT GCCGCCTCCGTCCGTACAAT
	ChCBF1	7	17908391	17908414	-	ATTGTACGGACGGAGGCGGC AAAA
Cas21aChCBF1_030	ChCBF2	4	6515261	6515284	+	TTTG CCGCCTCCGTCCGTACAATG
	ChCBF1	7	17908390	17908413	-	CATTGTACGGACGGAGGCGG CAAA

Finally, to contemplate intraspecific allelic variability in the design of sgRNAs for genome editing, I used ARES-GT in combination with the genome sequences available through the Arabidopsis 1001 genomes project (https://1001genomes.org/). ARES-GT can be used to design ecotype-specific targets taking advantage of polymorphic sequences in the different accessions. Good quality genome assemblies of seven *A*. *thaliana* accessions (*An-1*, *C24*, *Cvi*, *Eri*, *Kyo*, *Ler* and *Sha*) [13] were downloaded, and ARES-GT was used to design sgRNAs targeting CBF genes in each accession. As reflected in Table 4, the SNPs in *CBF* genes between the different accessions are responsible of the identification of different number of candidate sgRNAs that match several genes of the family, from 18 Cas9 candidates with *CBF* genes from *Kyo* genome to 11 Cas9 candidates with *CBF* genes from *Cvi* genome. The selection of CRISPR candidates with specific unique target (without offtargets) also varied between accessions (Table 4). I used each accession CBF genes as query for ARES-GT but using either the standar *Col-0* reference or the corresponding accession genome. Candidates only listed when *Col-0* is used as reference (*Col-0* exclusive) are false positives, as they have offtargets in the corresponding accession genome. The accession`s exclusive candidates would be false negatives, as they are discarded if *Col-0* is used but do not have offtargets in the corresponding accession genome (Table 4). Differences in the identification of offtargets also affects the selection of efficient candidates matching several CBF genes. For instance, candidate C24_CBF1_019 perfectly match C24_CBF1, C24_CBF2 and C24_CBF3 but has a possible offtarget (4 mismatches in distal sequence) in the chromosome 3 of *C24* genome, which is above offtarget thresholds in *Col-0* genome because of an extra mismatch in the proximal sequence (Table 5). In the other sense, Eri_Cas12aCBF1_017 is a candidate that perfectly match Eri_CBF1, Eri_CBF2 and Eri_CBF3 without offtargets in Eri genome, however it would be discarded because two offtargets are detected if *Col-0* genome is used (Table 5).

**Table 4:** Intraspecies variability effect in the number of Cas9 and Cas12a candidates targeting multiple or unique *AtCBF* genes. Sequence variability in the *CBF* genes from different *Arabidopsis thaliana* accessions change the number of candidates that can match multiple targets due to SNPs in the 20 nucleotides of the guide but also SNPs affecting PAM sequence. The use of the standard *Col-0* genome reference (TAIR v10) or the corresponding accession genome affects the identification of offtargets thus the correct identification of specific (unique) candidates matching only one *CBF* gene. The column “exclusive” indicates the number of specific candidates that are only listed when the corresponding reference genome is used.

*CBF* genes accession	Multiple Targets Candidates		Reference Genome	Unique Cas9 Candidates		Unique Cas12a Candidates	
	Cas9	Cas12a		Total	Exclusive	Total	Exclusive
*Col*	13	10	*Col*	96	-	34	-
*An-1*	13	9	*Col*	100	3	37	2
			*An-1*	105	8	41	6
*C24*	13	10	*Col*	100	4	33	2
			*C24*	101	5	31	0
*Cvi*	11	9	*Col*	102	6	34	3
			*Cvi*	107	11	37	6
*Eri*	13	10	*Col*	101	2	32	1
			*Eri*	101	2	31	0
*Kyo*	18	6	*Col*	99	8	32	2
			*Kyo*	103	12	33	3
*Ler*	13	10	*Col*	102	3	32	0
			*Ler*	105	6	34	2
*Sha*	13	10	*Col*	101	6	31	2
			*Sha*	102	7	31	2

**Table 5:** Intraspecies variability effect in the identification of targets and possible offtargets. For each example, upper file shows the targets and offtargets listed by ARES-GT (with thresholds L0 = 4 and L1 = 3) for each reference genome. SNPs differences between genomes that explain why some targets or offtargets are not detected are shown in lower file (separated by discontinuous line) as red boxes. Black boxes mark mismatches with candidates sequence.

Candidate ID						
*A*. *thaliana*	*Gene*	*chrom*	*start*	*end*	*sense*	*sequence*
C24_Cas21aCBF1_019	C24CBF2	C24_4	13745457	13745480	-	TCGGAGCCAAACATTTCAGA AAAA
	C24CBF3	C24_4	13748381	13748404	-	TCGGAGCCAAACATTTCAGA AAAA
	C24CBF1	C24_4	13751940	13751963	-	TCGGAGCCAAACATTTCAGA AAAA
	----	C24_3	4670219	4670243	+	TTTG TCTGAAATGTGCAGTTCCGA
	ColCBF3	Col_4	13018950	13018973	-	TCGGAGCCAAACATTTCAGA AAAA
	ColCBF1	Col_4	13022509	13022532	-	TCGGAGCCAAACATTTCAGA AAAA
	ColCBF2	Col_4	13016046	13016068	-	TCGGAGCCAAACATTTCAGA AAAG
	----	Col_3	4673610	4673633	+	TTTG TCTGAAAGGTGCAGTTCCGA
Eri_Cas12aCBF1_017	EriCBF2	Eri_4	12981374	12981397	-	AATCGGAGCCAAACATTTCA GAAA
	EriCBF3	Eri_4	12984307	12984330	-	AATCGGAGCCAAACATTTCA GAAA
	EriCBF1	Eri_4	12987866	12987889	-	AATCGGAGCCAAACATTTCA GAAA
	ColCBF2	Col_4	13016031	13016054	-	AATCGGAGCCAAACATTTCA GAAA
	ColCBF3	Col_4	13018948	13018971	-	AATCGGAGCCAAACATTTCA GAAA
	ColCBF1	Col_4	13022507	13022530	-	AATCGGAGCCAAACATTTCA GAAA
	----	Col_1	8279033	8279056	-	AATCAGAGCCTAACACTTCA AAAA
	----	Col_3	9399469	9399493	+	TTTA TGAAGTGTTTGGTTCCTATT
	----	Eri_1	8194484	8194507	-	AATTAGGGCCTAACACTTCA AAAA
	----	Eri_3	9400735	9400758	+	TTTA TGAAGTGTTTGGTTCCTTTT

## Discussion

Sequence similarity in gene families usually difficults the identification of CRISPR target candidates matching several member of the family and it requires manual time-consuming task. ARES-GT in addition of gene specific guide RNAs also evaluates which candidates match several query sequences. By selection of which sequences are included in the query file user has the maximal flexibility for working with complete families, subfamilies or a particular set of genes to find candidates specifically matching those genes. I have also shown how using ecotype-specific genomes can prevent the identification of false positive/negative candidates, which also apply to individual genomes taking into account polymorphisms.

ARES-GT is written in Python so can be used in any operative system and it has not high computational complexity so it is expected to work without problems with any processor. ARES-GT also has an option for working only with candidates matching several query sequences (option “–OR”) which reduce computer time to 15 min.

## Conclusion

In summary, I have shown how the architecture of the ARES-GT tool (i) allows the selection of candidate sgRNAs that match multiple input query sequences for simultaneous editing of several members of gene families; (ii) contemplates the use of unmapped contigs apart from complete genomes; and (iii) can be used for the design of ecotype-specific CRISPR targets. ARES-GT is available at GitHub (https://github.com/eugomin/ARES-GT.git).

## Supporting information

S1 File CBF genes. DNA sequences of all CBF genes used in this work.
(ZIP)
